# Texture Analysis of Food Samples Used for the Evaluation of Masticatory Function

**DOI:** 10.7759/cureus.58721

**Published:** 2024-04-22

**Authors:** Yuko Shikama, Kaede Matsuyama, Hiroko Kobayashi, Takahiro Suzuki, Aiji Sato-Boku, Motoko Takaoka, Yasuyuki Shibuya

**Affiliations:** 1 Oral and Maxillofacial Surgery, Nagoya City University Graduate School of Medical Sciences, Nagoya, JPN; 2 Anaesthesiology, Aichi Gakuin University, Nagoya, JPN; 3 Biosphere Sciences, Kobe College, School of Human Sciences, Nishinomiya, JPN

**Keywords:** cohesiveness, hardness, gumminess, masticatory function, texture analysis, food questionnaire survey

## Abstract

Introduction

Food questionnaire surveys are often used to evaluate masticatory function. In daily clinical practice in Japan, a survey is performed using a list of food groups suitable for the Japanese diet. The foods on the list were categorized into five food groups based on their mastication index. The patient's masticatory function is determined by the food groups that can be eaten. The masticatory index, which indicates chewability, was defined based on the percentage of 110 denture wearers who responded that they could eat food normally. A survey with this list is useful because of its simplicity; however, there is a lack of objective data on the physical properties of food samples. Consequently, to make the results of the food questionnaire survey more objective indicators, we performed a texture analysis of the food samples on the list.

Methods

We performed a texture analysis of 93 samples from 77 food items on the list. Compression tests were performed using a texture analyzer, and hardness, cohesiveness, adhesiveness, viscosity, and gumminess were calculated by a texture profile analysis.

Results

Even with the same ingredients, the results differed depending on the presence or absence of food skin, the direction of pressing (vertical or horizontal), cooking methods, and temperature differences. However, the masticatory index was negatively correlated with hardness (-0.4157, p<0.001) and gumminess which is determined as the product of hardness×cohesiveness (-0.4980, p<0.001).

Conclusion

This study suggests that the masticatory index indicating chewability may be related to the hardness and cohesiveness of food samples. Even for foods with the same hardness, the degree of difficulty in forming a food mass is expected to vary depending on differences in cohesiveness. Moreover, the presence or absence of food skin, the direction of food fibers, cooking methods, and temperature differences change the physical properties of the food. Therefore, the composition and structure of the foods or eating habits of patients should be taken into consideration when conducting a food questionnaire survey.

## Introduction

Mastication, to crush and mix food fragments, requires an appropriate occlusal relationship between the upper and lower jaws, a fitted tooth crown shape, and an accurate movement of the tongue with the buccal mucosa and the lips, which cooperates with jaw movements. In addition, saliva also plays an important role in bolus formation. These oral functions are greatly impaired by the loss of teeth, jawbone, and oral soft tissues, and most patients with oral tumors have severe trouble with mastication and bolus formation. In particular, segmental mandibulectomy causes anatomical distortion resulting in a functional deficit [1]. Radiotherapy to the oral cavity also reduces the secretory capacity of the salivary glands. These changes in oral function must be appropriately evaluated in clinical practice. Direct and indirect methods are used to evaluate masticatory function [2]. Indirect examination methods evaluate mastication by judging jaw movement, electromyography, the occlusal contact state, and occlusal force. In contrast, direct examination involves the subject actually chewing a masticatory sample (e.g., gum or gummy jelly) [3-5] and methods based on a food questionnaire survey.

Gum and gummy jelly are very useful because they can be objectively evaluated, but they are too hard to evaluate in patients with significant deterioration in the oral function. In fact, when the masticatory function after mandibular resection was measured with gummy jelly, most patients scored ≤2 out of a maximum score of 9 [4]. This was because the gummy jelly was so hard that the patient could not bite it off in multiple small pieces. Therefore, the test results may have underestimated the actual masticatory function of patients with oral dysfunction. On the contrary, even in the same patients, different results were obtained from a food questionnaire survey using the masticatory function evaluation table reported by Sato et al. which is often used in daily clinical practice in Japan [3,4,6-8]. The questionnaire with this table made it possible to evaluate the chewability of foods at levels 2, 3, and 4 of the five categories using the masticatory index described below. At levels 2, 3, and 4, there were foods that were neither too soft nor too hard, even in patients with oral dysfunction [8]. Ingredients that were equivalent to level 2 are, for example, sausages and hamburgers, etc., level 3 are bacon and bamboo shoots, etc., and level 4 are pickled radish and fresh abalone, etc. Therefore, the table by Sato et al. may be useful in evaluating the function of chewing such moderately hard foods by patients with oral dysfunction. On the other hand, there is a lack of objective data on the physical properties of the food samples in the table. In this table, the masticatory index is used as a measure of masticatory function; however, it was determined based on the percentage of 110 denture wearers who responded that they could eat the food normally in the study of Sato et al. The masticatory index is useful because of its simplicity, but it is not supported by objective data on the physical properties of foods. Consequently, to make the results of the food questionnaire survey more objective indicators, we performed a texture analysis of the food samples listed on the questionnaires to examine their physical properties.

## Materials and methods

Samples

Of the 100 items listed in the masticatory function evaluation table for complete dentures [6], we first excluded non-food items (i.e., cotton thread, mandarin orange bags, etc.). Moreover, we deleted food items that are not compatible with modern Japanese eating styles, such as *okoshi* (a type of Japanese cracker) or *amanatto* (a type of Japanese sweet), while we added new food items that suit our modern diet (e.g., avocado [9], croissant [10], etc.). Food items that were difficult to mold into the form for the measurement described below (e.g., peanuts, potato chips, lettuce, etc.) were also excluded. On the other hand, we used multiple types within the same category in some food items. For example, four types of gummy jelly and two types of jelly from different manufacturers were used for the measurement. It should be noted that Engelead jelly, which is a pharmaceutical, as defined by the Japan Consumer Affairs Agency, for patients with difficulty swallowing [11], was also employed. Additionally, two types of cheese (cream cheese and processed cheese) and two types of tofu (firm tofu and silken tofu) were included. In the same manner, baumkuchen and financier were included in the cakes. Finally, 77 food items that could be commercially available were used as samples (Table 1). Sample pieces were processed into cubes of 1.5×1.5×1.5 cm as a rule. If the thickness of the food is less than 1.5 cm, only the length and width were molded to 1.5×1.5 cm. Products that are difficult to mold, such as pudding, were measured as they were. Some food items were measured both raw and cooked. Some food items were measured both skinned and unskinned, such as grapes and pumpkins. Bread both with and without the crust was used. For some ingredients for which the lengths in the vertical and horizontal directions differed, measurements were taken both longitudinally and transversely. Finally, this study was conducted using 93 samples of 77 food items.

**Table 1:** List of measurement values for hardness, cohesiveness, adhesiveness, viscosity, and gumminess. Co., Ltd.: Company, Limited; Ltd.: Limited; Inc.: Incorporated

Food name	Cooking method	Press direction	Temperature during measurement	Product name	Manufacturer (distributor)	Hardness			Cohesiveness			Adhesiveness			Viscosity			Gumminess		
*Allium chinense*/Chinese onion	As it is	Nothing worth mentioning	Room temperature	Shakishaki shokkan no rakkyō	Iwashita Corporation	3217.983	±	420.278	0.995	±	0.007	1.991	±	1.607	5.824	±	1.911	4077.124	±	409.751
Avocado	As it is	Nothing worth mentioning	Room temperature	Avocado	Calavo Growers, Inc.	1234.046	±	179.515	0.871	±	0.222	54.304	±	9.163	68.306	±	9.621	681.181	±	153.170
Bacon	Boiled	Nothing worth mentioning	Room temperature	Burokkubēkon	Marudai Food Co., Ltd.	1650.065	±	273.679	0.995	±	0.007	0.193	±	0.161	1.450	±	1.033	1556.717	±	186.815
Bacon	Grilled	Nothing worth mentioning	Room temperature	Burokkubēkon	Marudai Food Co., Ltd.	2714.443	±	244.341	0.995	±	0.007	0.300	±	0.339	2.692	±	2.044	2779.123	±	199.621
Bamboo shoots	Boiled	Nothing worth mentioning	Room temperature	Take no kono mizuni	Marunaka Food Co., Ltd.	3628.228	±	674.431	1.293	±	0.214	2.641	±	0.739	17.995	±	4.341	4798.488	±	1411.786
Banana	As it is	Nothing worth mentioning	Room temperature	Dole Gokusen	Dole Japan, Inc.	1767.866	±	101.561	0.995	±	0.007	133.356	±	19.233	175.243	±	35.605	733.352	±	152.209
Baumkuchen	As it is	Nothing worth mentioning	Room temperature	Baumkuchen	Yamazaki Baking Co., Ltd.	433.081	±	74.936	0.765	±	0.076	1.119	±	0.480	5.392	±	2.203	347.279	±	65.238
Beef	Grilled	Nothing worth mentioning	Room temperature	Beef (filet)	Unknown	677.615	±	171.263	1.400	±	0.121	1.032	±	0.331	3.058	±	0.887	947.798	±	272.877
Biscuits	As it is	Nothing worth mentioning	Room temperature	Marie Biscuit	Morinaga ＆ Co., Ltd.	2924.414	±	264.666	1.465	±	0.153	0.005	±	0.002	0.179	±	0.009	3950.906	±	562.649
Slice bread (central part)	Not toasted	Nothing worth mentioning	Room temperature	Kin no shokupan	Musashino Foods Corporation	24.621	±	1.139	0.995	±	0.007	0.020	±	0.035	0.277	±	0.106	27.068	±	0.802
Slice bread (crust part)	Not toasted	Nothing worth mentioning	Room temperature	Kin no shokupan	Musashino Foods Corporation	57.589	±	8.396	1.003	±	0.048	0.004	±	0.002	0.158	±	0.024	57.986	±	8.174
Carrot	Boiled	Nothing worth mentioning	Room temperature	Carrot	Unknown	635.924	±	72.357	0.452	±	0.109	3.260	±	0.303	5.769	±	0.556	310.257	±	100.967
Castella	As it is	Nothing worth mentioning	Room temperature	Kokutō suzu kasutera	Moheji Co., Ltd.	485.557	±	30.365	0.995	±	0.007	0.011	±	0.004	0.248	±	0.052	474.341	±	26.331
Chicken	Boiled	Nothing worth mentioning	Room temperature	Prima salad chicken	Prima Meat Packers, Ltd.	1243.383	±	232.469	1.232	±	0.161	6.166	±	5.088	13.570	±	5.175	1554.928	±	435.000
Chikuwa	As it is	Nothing worth mentioning	Room temperature	Kiwami no nama chikuwa	Maruha Nichiro Corporation	308.979	±	138.705	1.201	±	0.117	0.012	±	0.002	0.217	±	0.039	390.642	±	214.087
Firm tofu	Boiled	Nothing worth mentioning	Room temperature	Momen Tofu	Satonoyukisyokuhin Co., Ltd.	177.050	±	8.023	1.090	±	0.015	2.573	±	0.457	4.779	±	0.522	193.549	±	6.697
Firm tofu	Unboiled	Nothing worth mentioning	Room temperature	Momen Tofu	Satonoyukisyokuhin Co., Ltd.	134.434	±	15.100	1.100	±	0.010	2.479	±	1.107	4.240	±	1.036	148.571	±	15.014
Cream cheese	As it is	Nothing worth mentioning	17.5℃	Kiri creamy potion	Itoham Foods Inc.	398.646	±	59.853	1.518	±	0.065	66.691	±	10.084	100.655	±	9.440	611.047	±	34.249
Croissant	As it is	Nothing worth mentioning	Room temperature	Mini croissant	Yamazaki Baking Co., Ltd.	49.087	±	10.595	1.023	±	0.051	0.004	±	0.002	0.166	±	0.009	50.317	±	9.663
Daifuku/Japanese sweet	As it is	Nothing worth mentioning	Room temperature	Kine-tsuki daifuku	Yanouseika Co., Ltd.	435.936	±	55.370	2.002	±	0.259	0.007	±	0.002	0.200	±	0.014	873.926	±	169.082
Donut	As it is	Nothing worth mentioning	Room temperature	Oak Hills King donut	Marunaka Confectionery Co., Ltd.	389.497	±	37.621	0.995	±	0.007	0.600	±	0.206	2.551	±	0.700	337.545	±	64.366
Dorayaki	As it is	Nothing worth mentioning	Room temperature	Kuri-iri dora-yaki	Kotobuki Seika Co., Ltd.	144.910	±	29.434	1.203	±	0.075	1.435	±	0.787	3.535	±	1.746	180.385	±	51.672
Eggplant	Boiled	Nothing worth mentioning	Room temperature	Eggplant	Cico Mart Co., Ltd.	535.941	±	96.029	1.938	±	0.295	14.837	±	6.969	18.599	±	2.283	1072.938	±	159.352
Financier	As it is	Nothing worth mentioning	Room temperature	Financier	Seven & i Holdings Co., Ltd.	524.403	±	48.438	1.017	±	0.075	0.570	±	0.226	2.605	±	0.661	558.827	±	52.148
Fish sausage	As it is	Lateral press	Room temperature	SausageMS 3 bundles	Toyo Suisan Co., Ltd.	440.972	±	18.840	0.984	±	0.002	4.407	±	0.608	16.415	±	1.627	434.273	±	19.538
Fish sausage	As it is	Vertical press	Room temperature	SausageMS 3 bundles	Toyo Suisan Co., Ltd.	649.588	±	17.514	0.928	±	0.013	3.622	±	0.676	21.373	±	4.605	602.735	±	9.442
Fried tofu	As it is	From inner side	Room temperature	Atsu-age	Kurita-jyunpaku Co., Ltd.	233.279	±	25.116	0.895	±	0.051	10.807	±	1.187	4.771	±	0.789	210.033	±	34.301
Fried tofu	As it is	From outer side	Room temperature	Atsu-age	Kurita-jyunpaku Co., Ltd.	144.656	±	35.331	1.065	±	0.031	0.140	±	0.122	0.531	±	0.382	153.403	±	34.968
Ganmodoki	Boiled	Nothing worth mentioning	Room temperature	Oden ganmo	Kanesada Co., Ltd.	161.352	±	51.714	1.137	±	0.092	0.425	±	0.209	1.716	±	0.692	183.201	±	71.182
Grape 1 (skinned)	As it is	Nothing worth mentioning	Room temperature	Pione	Unknown	330.317	±	57.041	2.162	±	0.100	1.546	±	0.790	3.487	±	1.035	725.693	±	119.075
Grape 1 (unskinned)	As it is	Nothing worth mentioning	Room temperature	Pione	Unknown	1004.474	±	86.112	1.001	±	0.034	0.015	±	0.016	0.412	±	0.291	1000.218	±	73.755
Grape 2 (skinned)	As it is	Nothing worth mentioning	Room temperature	Kyoho (with seeds)	JA-Fukuoka Yame	443.781	±	81.573	2.368	±	0.122	3.387	±	0.818	4.371	±	1.066	1033.950	±	201.503
Grape 2 (unskinned)	As it is	Nothing worth mentioning	Room temperature	Kyoho (with seeds)	JA-Fukuoka Yame	761.227	±	58.616	1.029	±	0.023	0.057	±	0.108	0.695	±	1.168	785.218	±	78.254
Green soybean/edamame (frozen food)	Thawing	Nothing worth mentioning	Room temperature	Amami to yutakana fūmi edamame	Maruha Nichiro Corporation	3407.785	±	266.639	0.494	±	0.099	3.263	±	0.895	3.427	±	0.614	1733.557	±	370.198
Grilled eel	As it is	Nothing worth mentioning	Room temperature	Sumibi-teyaki unagi	Blue Ocean Co., Ltd.	686.058	±	113.162	1.395	±	0.059	0.937	±	0.566	3.433	±	1.723	991.353	±	186.782
Gummy 1	As it is	Nothing worth mentioning	Room temperature	TOUGH	Kabaya Foods Corporation	2600.641	±	167.364	1.036	±	0.017	0.004	±	0.002	0.171	±	0.018	2777.537	±	149.646
Gummy 2	As it is	Nothing worth mentioning	Room temperature	MEIJI juicy gummy	Meiji Co., Ltd.	2004.413	±	181.187	1.100	±	0.030	1.513	±	0.384	6.237	±	1.238	2246.169	±	236.302
Gummy 3	As it is	Nothing worth mentioning	Room temperature	DHC supplement	DHC Corporation	846.681	±	93.673	1.013	±	0.003	0.036	±	0.013	0.548	±	0.131	810.282	±	116.293
Gummy 4	As it is	Nothing worth mentioning	Room temperature	Kororo	UHA Mikakuto Co., Ltd.	458.927	±	62.503	1.072	±	0.041	0.229	±	0.017	1.059	±	0.128	500.652	±	90.482
Gyoza	Grilled	Nothing worth mentioning	65℃	Usukawa hamamatsu gyoza	Marumatsu Co., Ltd.	96.293	±	18.042	1.263	±	0.109	0.219	±	0.463	0.613	±	0.874	112.989	±	24.045
Gyoza	Grilled	Nothing worth mentioning	Room temperature	Usukawa hamamatsu gyoza	Marumatsu Co., Ltd.	88.249	±	12.929	1.193	±	0.121	0.305	±	0.309	1.021	±	0.638	100.911	±	35.032
Hamburg steak	Grilled	Nothing worth mentioning	65℃	Kinoko-iri hanbāgu wafū sōsu	Prima Meat Packers, Ltd.	669.828	±	105.775	1.218	±	0.135	14.293	±	2.359	20.231	±	5.197	737.075	±	68.073
Hamburg steak	Grilled	Nothing worth mentioning	Room temperature	Kinoko-iri hanbāgu wafū sōsu	Prima Meat Packers, Ltd.	757.319	±	121.249	1.023	±	0.029	2.780	±	1.037	7.465	±	2.879	770.818	±	113.119
Hanpen	Boiled	Nothing worth mentioning	Room temperature	Hanpen	Kibun Foods Inc.	204.255	±	21.074	1.060	±	0.062	11.491	±	7.912	12.626	±	4.473	218.054	±	20.384
Hanpen	Unboiled	Nothing worth mentioning	Room temperature	Hanpen	Kibun Foods Inc.	237.086	±	43.342	1.029	±	0.028	10.990	±	2.584	20.695	±	6.000	244.206	±	47.688
Jelly 1	As it is	Nothing worth mentioning	Room temperature	Kon'nyakubatake	Mannan Life Co., Ltd.	279.292	±	36.344	1.089	±	0.191	7.818	±	1.627	4.350	±	0.796	282.439	±	66.768
Jelly 2 (pharmaceuticals)	As it is	Nothing worth mentioning	Room temperature	Engelead	Otsuka Pharmaceutical Co., Ltd.	78.662	±	0.827	1.146	±	0.048	6.575	±	0.795	6.575	±	0.795	88.696	±	2.338
Kamaboko	As it is	Nothing worth mentioning	17.5℃	Bettora kamaboko	Bettora Kamaboko Co., Ltd.	764.405	±	121.821	0.995	±	0.008	8.223	±	1.390	23.867	±	2.920	766.401	±	116.909
Kneaded yokan/Japanese sweet	As it is	Nothing worth mentioning	Room temperature	Neri-yō kan	Imuraya Group Co., Ltd.	762.809	±	40.427	0.592	±	0.081	74.237	±	11.334	63.602	±	4.839	447.053	±	57.521
Konnyaku	Boiled	Nothing worth mentioning	Room temperature	Ita kon'nyaku	Uesugisyokuhin Co., Ltd.	445.119	±	91.092	1.141	±	0.074	2.921	±	0.719	7.213	±	2.111	496.846	±	129.888
Konnyaku	Unboiled	Nothing worth mentioning	Room temperature	Ita kon'nyaku	Uesugisyokuhin Co., Ltd.	670.218	±	189.635	0.982	±	0.016	4.486	±	1.388	11.752	±	2.775	667.187	±	198.394
Mandarin orange	As it is	Nothing worth mentioning	Room temperature	Kōchi ken-san hausu mikan	JA-Kochi	589.730	±	109.275	1.485	±	0.047	1.137	±	0.626	2.975	±	1.246	882.798	±	150.680
Marshmallow	As it is	Nothing worth mentioning	Room temperature	White marshmallow	Eiwa Confectionary Co., Ltd.	215.419	±	12.955	1.054	±	0.013	0.004	±	0.001	0.214	±	0.023	228.357	±	10.990
Meatball	As it is	Nothing worth mentioning	Room temperature	Hokkaidō-san toriniku no mītobōru	NH Foods Ltd.	428.868	±	52.859	1.159	±	0.066	6.511	±	2.928	11.125	±	4.040	483.225	±	47.349
Monaka/Japanese sweet	As it is	Nothing worth mentioning	Room temperature	Kuro goma monaka	Shiawasedo Co., Ltd.	1132.186	±	47.337	0.349	±	0.048	43.760	±	25.968	23.268	±	6.827	956.441	±	347.622
Octopus legs	Boiled	Nothing worth mentioning	Room temperature	Octopus (made in Morocco)	Unknown	706.716	±	333.198	0.995	±	0.007	2.563	±	3.146	8.418	±	7.200	742.270	±	307.206
Peach	As it is	Nothing worth mentioning	Room temperature	Peach	Unknown	429.116	±	73.821	0.619	±	0.122	27.721	±	13.790	20.000	±	6.639	389.503	±	165.928
Pear	As it is	Nothing worth mentioning	Room temperature	Hōsui nashi	Unknown	3627.360	±	613.935	1.170	±	0.215	6.904	±	3.440	59.850	±	32.698	4085.506	±	249.582
Persimmon	As it is	Nothing worth mentioning	Room temperature	Wakayama ken-san tane nashi kaki	Unknown	1257.522	±	276.107	0.678	±	0.107	58.924	±	16.177	45.259	±	12.244	729.051	±	206.950
Pickled radish	As it is	Nothing worth mentioning	Room temperature	Kokusan amakuchi takuan Ippon-dzuke	Ag Link Co., Ltd.	3709.298	±	1658.686	1.144	±	0.397	43.498	±	42.312	47.373	±	37.359	3870.602	±	794.319
Pineapple	As it is	Nothing worth mentioning	Room temperature	Suu~ītio painburokku	Dole Japan, Inc.	1625.078	±	138.691	0.589	±	0.097	21.661	±	11.364	21.046	±	1.161	1153.772	±	305.112
Potato	Boiled	Nothing worth mentioning	Room temperature	May queen	Unknown	1251.186	±	232.954	0.356	±	0.086	26.801	±	9.397	30.073	±	10.652	957.556	±	119.393
Processed cheese	As it is	Nothing worth mentioning	17.5℃	Eating Milk Baby	Marinfood Co., Ltd.	1358.537	±	106.710	1.113	±	0.084	83.548	±	16.198	83.548	±	16.198	1594.130	±	68.616
Pudding	As it is	Nothing worth mentioning	15.5℃	Putchin purin	Ezaki Glico Co., Ltd.	65.684	±	6.239	0.878	±	0.129	9.644	±	1.295	3.351	±	0.410	38.555	±	8.951
Pumpkins	Boiled	From inner side	Room temperature	Kabochani	Fujicco Co., Ltd.	947.227	±	174.789	0.826	±	0.053	20.597	±	2.706	14.177	±	3.029	1006.983	±	217.697
Pumpkins	Boiled	From outer side	Room temperature	Kabochani	Fujicco Co., Ltd.	1440.499	±	518.308	0.849	±	0.054	1.932	±	1.080	11.103	±	3.191	1386.305	±	423.533
Radish	Boiled	Nothing worth mentioning	Room temperature	Daikon	Unknown	1239.445	±	97.098	0.466	±	0.086	205.569	±	10.906	7.299	±	0.976	762.381	±	246.224
Raisins	As it is	Nothing worth mentioning	Room temperature	Seika-yō kajitsu rēzun	Kyoritsu Foods Co., Ltd.	171.913	±	52.909	0.995	±	0.007	0.124	±	0.059	1.064	±	0.320	187.291	±	54.864
Ripe figs	As it is	Nothing worth mentioning	Room temperature	Ripe figs	Unknown	179.728	±	62.456	0.995	±	0.007	18.364	±	8.422	21.719	±	6.393	175.612	±	79.802
Sausage 1	Boiled	Nothing worth mentioning	Room temperature	Kawa nashi ara-biki pōkuuin'nā	Prima Meat Packers, Ltd.	1040.345	±	59.040	1.010	±	0.010	0.489	±	0.373	3.509	±	1.531	1061.598	±	58.471
Sausage 1	As it is	Nothing worth mentioning	Room temperature	Kawa nashi ara-biki pōkuuin'nā	Prima Meat Packers, Ltd.	1081.516	±	76.921	1.061	±	0.026	1.006	±	0.273	6.710	±	1.574	1155.597	±	64.604
Sausage 2	Boiled	Lateral press	Room temperature	Paritto shokkan no ara-biki uin'nā	Itoham Foods Inc.	626.263	±	45.197	1.065	±	0.018	0.829	±	0.447	4.640	±	1.789	674.209	±	57.169
Sausage 2	Boiled	Vertical press	Room temperature	Paritto shokkan no ara-biki uin'nā	Itoham Foods Inc.	1529.341	±	112.666	0.928	±	0.041	0.561	±	0.421	3.287	±	2.338	1456.772	±	48.616
Scallop adductor muscle	As it is	Nothing worth mentioning	Room temperature	Hotate kaibashira mizuni	Nissui Corporation	341.503	±	59.923	0.964	±	0.101	0.939	±	0.393	3.087	±	1.536	333.825	±	44.979
Shiitake mushrooms	Boiled	Nothing worth mentioning	Room temperature	Hyōgo Tanba-san	Unknown	250.571	±	36.115	1.299	±	0.212	2.849	±	1.115	12.631	±	5.584	329.035	±	90.740
Shrimp tempura	As it is	Nothing worth mentioning	Room temperature	Shrimp tempura	Unknown	285.824	±	48.161	1.374	±	0.077	0.104	±	0.056	0.657	±	0.463	445.291	±	159.712
Silk tofu	Boiled	Nothing worth mentioning	Room temperature	Kinu	Yamami Company	130.202	±	12.234	1.023	±	0.018	6.416	±	0.686	5.505	±	0.647	134.984	±	11.666
Silk tofu	Unboiled	Nothing worth mentioning	Room temperature	Kinu	Yamami Company	104.631	±	8.715	1.042	±	0.055	5.957	±	1.566	5.080	±	0.661	108.478	±	11.225
Soybeans	Boiled	Nothing worth mentioning	Room temperature	Konomama tsukaeru daizu mizuni	Fujicco Co., Ltd.	631.852	±	95.435	0.866	±	0.089	0.107	±	0.020	1.165	±	0.291	582.595	±	121.514
Steamed cake	As it is	Nothing worth mentioning	Room temperature	Hokkaidō chīzu mushi kēki	Yamazaki Baking Co., Ltd.	72.069	±	3.084	1.048	±	0.019	2.789	±	0.569	3.982	±	1.629	76.466	±	5.427
Sweet chestnut	As it is	Nothing worth mentioning	Room temperature	Kuri honrai no hokuhoku shokkan yūki muki amaguri	Maruseishoji Co., Ltd.	4639.171	±	1012.834	0.579	±	0.159	0.042	±	0.074	0.722	±	0.860	2544.003	±	318.642
Sweet potato	Boiled	Nothing worth mentioning	Room temperature	Satsumaimo no amani	Fujicco Co., Ltd.	1079.165	±	65.047	0.781	±	0.084	53.361	±	22.623	82.778	±	16.292	883.267	±	204.145
Sword tip squid	As it is	Nothing worth mentioning	Room temperature	Nagasaki ken-san kensaki ika	Unknown	1680.342	±	808.101	1.138	±	0.569	17.927	±	3.996	21.575	±	8.579	1666.609	±	572.008
Takoyaki	Warmed	Nothing worth mentioning	80℃	Takoyaki	Shosan Shoji Co., Ltd.	90.586	±	18.845	1.096	±	0.032	7.109	±	6.691	8.360	±	6.437	100.373	±	18.305
Taro	Boiled	Nothing worth mentioning	Room temperature	Marugoto ara-muki satoimo	Shimizu Products Co., Ltd.	1755.900	±	444.979	0.385	±	0.130	48.988	±	26.890	46.792	±	15.517	1162.553	±	554.692
Thick fried egg	As it is	Nothing worth mentioning	Room temperature	Kireteru atsuyakitamago	Isedelica Co., Ltd.	220.013	±	16.113	0.930	±	0.034	1.209	±	0.357	4.865	±	1.102	204.780	±	18.262
Three-color dumpling/Japanese sweets	As it is	Nothing worth mentioning	Room temperature	Otsukimi san-shoku dango	Yamazaki Baking Co., Ltd.	88.758	±	9.350	1.037	±	0.059	185.177	±	50.257	61.665	±	10.943	96.620	±	14.350
Tomato	As it is	Nothing worth mentioning	Room temperature	Tomato	Unknown	689.947	±	269.201	0.995	±	0.007	8.581	±	5.273	27.230	±	16.241	795.790	±	586.613
Tsukune	Boiled	Nothing worth mentioning	Room temperature	Yuzu nama tsukune	Hirao Co., Ltd.	401.278	±	91.524	1.124	±	0.025	0.171	±	0.233	1.246	±	1.116	449.096	±	98.569
Tuna sashimi	As it is	Nothing worth mentioning	17.5℃	Shizuoka-san maguro	Cico Mart Co., Ltd.	826.282	±	165.853	1.068	±	0.097	54.246	±	4.784	24.314	±	2.779	858.697	±	135.385
Turnip pickle	As it is	Lateral press	Room temperature	Hidatakayama akakabura-dzuke	Tsukemono-urata Co., Ltd.	590.052	±	270.595	1.420	±	0.491	4.278	±	1.792	9.016	±	3.098	1106.850	±	1212.502
Turnip pickle	As it is	Vertical press	Room temperature	Hidatakayama akakabura-dzuke	Tsukemono-urata Co., Ltd.	2178.565	±	895.955	1.429	±	0.395	4.148	±	1.523	10.632	±	2.856	3051.185	±	1514.603
White leek	As it is	Nothing worth mentioning	Room temperature	Hokkaidō-san Nakamura-san no shiro negi	Unknown	1729.054	±	269.884	0.995	±	0.007	0.037	±	0.036	0.558	±	0.391	1853.312	±	362.179

Cooking methods

Samples such as frozen foods were heated using a water oven, AX-GA1-W (Sharp Corporation, Japan). The water oven was set to the microwave function (power setting: 600 W). During heating, food samples were placed on a dessert plate (diameter: 19.5 cm) and lightly covered with a wrap film for food packaging, Dia Wrap Eco Pita! (Mitsubishi Plastics Co., Ltd., Japan). After heating, a stick thermometer, digital thermometer TT-533 (Tanita Co., Ltd., Japan), was used to insert the temperature part (tip 20 mm) into the sample, and the temperature was measured when the displayed temperature stabilized. For samples normally eaten at room temperature, the temperature was finally adjusted to 23±1°C. On the other hand, the actual temperature was considered when eating. For example, the temperature of dumplings and Hamburg steaks was set at 65°C, and the temperature of *takoyaki* was set at 80°C.

In the cases of grilling and boiling, we used an IH cooker, KZ-PH30P (Panasonic, Japan), with a medium heat setting of 700 W. A frying pan (diameter: 26 cm, depth: 4 cm) was used for grilling. Taking into account the thermal conductivity of the frying pan, the sample was added 30 seconds after the ignition of the IH cooker. The food samples were cooked according to general cooking methods. For example, bacon was heated over medium heat for 30 seconds on each side. In the case of boiling, 1 liter of distilled water was placed in a pot (diameter: 16 cm, depth: 7 cm), and the sample was placed in the pot after boiling. However, if there was a description of the cooking method on the package, it was followed. For example, *tsukune* was boiled in a pot containing 400 ml of water for 10 minutes.

Foods that were likely to lose their shape after cooking were molded into cubes of 1.5 cm after cooking, but the other samples were molded before cooking. For example, silken tofu, firm tofu, *hanpen*, and *konnyaku* were first cut into large pieces, then boiled, and finally shaped into 1.5 cm^2^ cubes. The sizes of these samples before boiling were as follows: silken tofu (length 7.5 cm, width 7.5 cm, thickness 2.5 cm); firm tofu (length 8.5 cm, width 5.5 cm, thickness 3.2 cm); *hanpen* (length 11.0 cm, width 11.0 cm, thickness 1.5 cm); and *konnyaku* (length 13.5 cm, width 7.0 cm, thickness 2.0 cm). The following samples were molded before cooking, but the boiling time was varied according to the ease of boiling: eggplant (one minute); bacon (five minutes), carrot (30 minutes), radish (20 minutes), and potato (10 minutes).

Measurement of food texture

**Figure 1:**
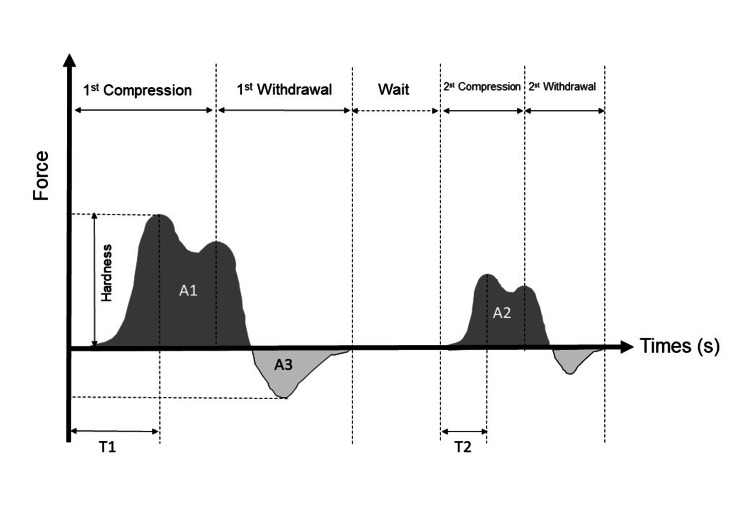
Illustration of the texture profile analysis method. Hardness is the height of the first peak. Cohesiveness is calculated by A2/A1. Adhesiveness is the area of A3. Viscosity is the height of the negative peak. Elasticity is calculated by T2/T1. S: second; A1: the area of the first positive peak; A2: the area of the second positive peak; A3: the area of the negative peak immediately after the first positive peak; T1: the time from start to peak of the first positive peak; T2: the time from start to peak of the second positive peak

A compression test was performed using a texture analyzer, TA.XTplusC (Eiko Seiki Co., Ltd., Japan), and the following items (items 1-5) were calculated according to the texture profile analysis method [12]: (1) hardness (defined as the force necessary to achieve a given deformation), (2) cohesiveness (defined as the strength of the internal bonds that make up the body of the product), (3) adhesiveness (defined as the work necessary to overcome the attractive forces between the surface of the food and the surface of other materials with which the food comes in contact), (4) viscosity (defined as the flow rate per unit force), and (5) elasticity (defined as the rate at which a deformed material returns to its undeformed condition after the deforming force is removed). Additionally, gumminess (the energy required to disintegrate a semi-solid food product to a state ready for swallowing) was determined as the product of hardness×cohesiveness, and chewiness (the energy required to masticate a solid food product to a state ready for swallowing) was determined as the product of hardness×cohesiveness×elasticity. As shown in Figure 1, hardness is the height of the first peak, cohesiveness is the area of the second positive peak divided by the area of the first positive peak (A2/A1), adhesiveness is the area of the negative peak immediately after the first positive peak (A3), viscosity is the height of the negative peak, and elasticity is the time from start to peak of the second positive peak divided by the time from start to peak of the first positive peak (T2/T1).

Measurement conditions such as the plunger size and compression speed were specified in detail. A cylindrical probe (diameter: 20 mm) was used, assuming clenching of the molars. Compression was performed twice, and the compression speed was set to 2.00 mm/s. The distance the probe moved after contacting the sample was set to 5 mm. The measurement was repeated eight times per sample type, and the maximum and minimum data were deleted. The remaining six measurements were used to calculate the mean values and standard deviations for hardness, cohesiveness, adhesiveness, and elasticity.

Statistical analysis

All statistical analyses were performed using Excel Statistics, Version 3.2 (Social Information Service Co., Ltd., Tokyo, Japan), and the Spearman correlation coefficient (r) was calculated. P-values of <0.05 were considered to indicate a statistically significant difference.

## Results

The hardness, cohesiveness, adhesiveness, and viscosity measurements are shown in Table 1. The maximum hardness was 4639.171 g (sweet chestnut). This was followed by pickled radish, bamboo shoots, pear, green soybeans (frozen food), and *Allium chinense *(Chinese onion). In contrast, the minimum hardness was 24.621 g (the central part of untoasted sliced bread), and croissant and sliced bread with crust (untoasted) also had low hardness values. The average hardness value was 918.926 g. Regarding cohesiveness, the maximum value was 2.368 (skinned Kyoho grape), and skinned Pione grapes and daifuku (Japanese sweet) also had high values, while the minimum value was 0.349 (monaka (Japanese sweet)), and boiled May queen potato and boiled taro also had also low values. The average value of cohesiveness was 1.057. The maximum adhesiveness value was 205.569 (boiled radish) and the minimum was 0.004 (marshmallow, TOUGH® gummy, croissant, and untoasted sliced bread crusts). The average adhesiveness value was 15.644. The maximum viscosity was 175.243 (bananas) and the minimum was 0.158 (untoasted sliced bread crusts). The average viscosity value was 15.572. Elasticity values were approximately 1.0 for almost all food items, and there was no significant difference; therefore, the gumminess values (hardness×cohesiveness) and chewiness values (hardness×cohesiveness×elasticity) were almost the same. The maximum gumminess was 4798.488 (boiled bamboo shoots), followed by pear and *Allium chinense* (Chinese onion). The minimum gumminess was 27.068 (central part of untoasted sliced bread), followed by pudding crust, croissant, and untoasted sliced bread. The average gumminess value was 931.156.

In this research, we occasionally found that even with the same ingredients, the results differed depending on the presence or absence of the skin, the direction of pressing (vertical or horizontal), the cooking methods, and the difference in temperature. For example, in the case of Kyoho grapes, differences were observed in all measured values, depending on the presence or absence of the skin. Skinless Kyoho grapes (with seeds) had the following values: hardness, 443.781; cohesiveness, 2.368; adhesiveness, 3.387; and viscosity, 4.371. In contrast, Kyoho grapes with skin (with seeds) had the following values: hardness, 761.227; cohesiveness, 1.029; adhesiveness, 0.057; and viscosity, 0.695. In the case of white bread, the crust remaining slice was harder, but there were no significant differences in other indicators. However, differences were only found in the hardness between the vertical and horizontal directions in which the samples had different internal fiber orientations. A typical example was pickled turnip. A vertical push produced the following values: hardness, 2178.565; cohesiveness, 1.429; adhesiveness, 4.148; and viscosity, 10.632. In contrast, a horizontal push produced the following values: hardness, 590.052; cohesiveness, 1.420; adhesiveness, 4.278; and viscosity, 9.632. Regarding cooking methods, many ingredients were found to be softer when boiled than when raw, but both silken and firm tofu became harder when boiled. In comparison to baking and boiling, baked bacon was harder than boiled bacon. In terms of the food temperature at the time of measurement, the adhesiveness and viscosity of the Hamburg steak were extremely high at higher temperatures, but the hardness remained almost the same.

**Figure 2:**
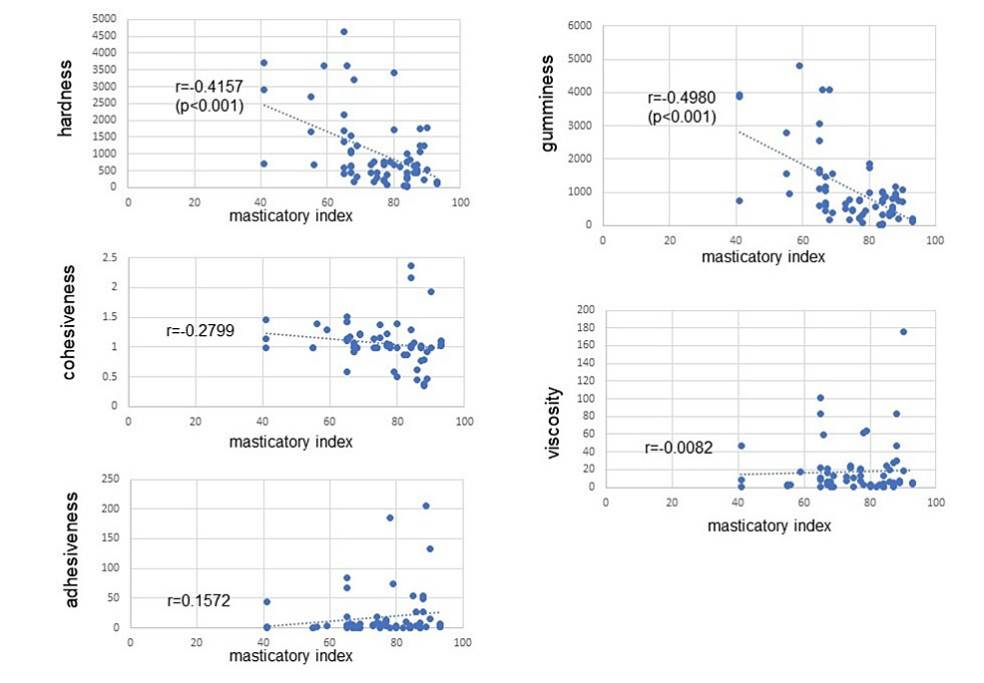
Correlation between the masticatory index and each texture analysis result.

In the table for evaluating the masticatory function for complete dentures [6], the masticatory index of the food was determined based on the percentage of 110 denture wearers who responded that they could eat the food normally. We investigated a Spearman correlation coefficient between this quantitative index and the data of this study. The correlation coefficients with the masticatory index were as follows: hardness, -0.4157 (p<0.001); cohesiveness, -0.2799; adhesiveness, 0.1572; and viscosity, -0.0082. The masticatory index showed a negative significant correlation with hardness (-0.4157, p<0.001) and gumminess which is determined as the product of hardness×cohesiveness (-0.4980, p<0.001) (Figure 2).

## Discussion

From ingestion to swallowing, the processes of biting, crushing, mixing, and bolus formation of foods are necessary. However, these oral functions are greatly reduced in patients with oral cancer. One of the reasons is that surgical resection is the standard treatment for oral cancer [13]. If the jawbone is surgically resected, hard reconstruction should be performed using a fibular flap or reconstruction plate; furthermore, a jaw prosthesis using dental implants may be needed. However, it has been shown that the improvement in masticatory function is limited in such case [3,4,8,14]. Additionally, radiotherapy causes a dry mouth, which is one of the main oral function problems, since sufficient saliva is required for bolus formation. Consequently, it is necessary to accurately evaluate the oral functions, including the masticatory function, after treatment for oral cancer. Thus far, we have evaluated the oral function using gum, gummy jelly, and food questionnaires. Gum and gummy jelly are very reliable because they can be objectively evaluated, but they are too hard to evaluate in patients with significant deterioration in the oral function. In other words, the food questionnaire survey, whose items include foods with a variety of characteristics, including hardness, could allow a more realistic and detailed assessment of the masticatory function in patients with oral dysfunction. However, there is the possibility that the results of the food questionnaire survey may change depending on the subjectivity of the patient. In addition, objective data on the physical properties of food samples described in the questionnaires are lacking. Therefore, in this study, to perform a more objective evaluation, we analyzed the texture of the food samples of the questionnaire and examined their physical properties.

We found that the physical properties of individual foods differed depending on the presence or absence of food skin, the direction of food fibers, the cooking methods, and the temperature. For example, all the values of all measured items differed depending on the presence or absence of grape skin. The skin protects the fragile and soft pulp, and it is natural that the presence of skin would increase the hardness. In contrast, the skin itself reduced the values of cohesiveness, adhesiveness, and viscosity. It can be considered that the grape skin prevented the pieces of pulp from mixing and adhering to each other. Accordingly, the grape skin should be a suppressor of bolus formation. Regarding bread, each numerical value of texture measurement was different depending on the structure and material of the breads [15]. In this study, when comparing breads with and without crust, the hardness of the bread with crust was more than twice as high, but the cohesiveness, adhesiveness, and viscosity were almost the same. Unlike grape skins, bread crusts probably do not have the effect of preventing food particles from mixing or sticking to each other. Differences in hardness were also observed according to the orientation of the internal fiber whether vertical or horizontal, even for the same food material. Foods with fibers, such as vegetable stems, may become harder if the chewing direction matches the direction of the fibers. A typical example of such cases in this study was pickled turnip; however, bolus formation after fine chewing is thought to be independent of the direction of the fiber, because there were no differences in cohesiveness, adhesiveness, and viscosity. Considering the cooking method, when comparing the methods of baking and boiling bacon, the former was found to be harder. This is thought to be due to a change in the amount of water contained in the ingredient. It was considered that baking rather than boiling tended to decrease the amount of moisture contained in the ingredients. In the case of tofu, both the silken and the firm tofu became slightly firmer after boiling. Tofu is made by hardening soy milk with calcium salt. It is known that heating promotes further bonding between soy protein and calcium ions, and as a result, the hardness should increase even after the protein has returned to room temperature. However, even when Hamburg steak was measured at a high temperature (65℃), the hardness did not change much in comparison to room temperature. In contrast, the adhesiveness and the viscosity of the Hamburg steak increased significantly at a high temperature. It is thought to be due to the oil becoming lubricated by heating and the crumbly texture of the ingredients being reduced. On the basis of these results, it is considered necessary to confirm the composition and structure of the foods or eating habits when conducting a questionnaire survey.

In this study, the masticatory index described in the complete denture masticatory function evaluation table by Sato et al. showed a significantly negative correlation with both hardness and gumminess, and gumminess resulted in a higher correlation coefficient value. Gumminess is defined as the energy required to break down a semi-solid food into fragments until it is ready to swallow [12], and its value is calculated by the formula of hardness×cohesiveness [16]. Since the masticatory index by Sato et al. has been useful for evaluating masticatory function, cohesiveness is considered to be as important as hardness when evaluating masticatory function. The cohesiveness was shown to indicate the strength of internal bonds that make up the body of food and the degree to which a food can be deformed before it ruptures (breaks) [16]. Yoshimine et al. demonstrated that cohesiveness should be evaluated as the index of the ability to dilute a bolus with saliva and also stated that multiple parameters should be measured for a comprehensive assessment of mastication [17]. According to the results of this study, even with foods of almost the same hardness, for example, boiled eggplant (the masticatory index is 90 and hardness was 535.941 g) had high cohesiveness (1.938) and was likely to form a bolus, while fish sausage (the masticatory index is 67 and hardness was 440.972) had low cohesiveness (0.984) and would be difficult to become a bolus. It may be difficult to form a bolus from foods with low cohesiveness, especially for people with low saliva production. Engelead is a jelly product approved as a food for special dietary use, "Food for persons who have difficulty swallowing: approval standard I," as stipulated by the Consumer Affairs Agency [11]. Among the food samples in this study, this jelly was the sixth softest (78.662 g) and had the 23rd highest cohesiveness (1.146). Therefore, from these results, it can be reasonably said that this jelly is suitable for people who have difficulty swallowing. To evaluate the actual mastication in detail, it is important to also investigate the cohesiveness of food samples in addition to their hardness. However, only 93 food samples were examined in this study, which is not a sufficient number. Further research is needed in the future to increase the number of subjects.

## Conclusions

This study suggests that chewability may be related to the hardness and cohesiveness of food samples. Even for foods with the same hardness, the degree of difficulty in forming a food mass is expected to vary depending on differences in cohesiveness. Moreover, the presence or absence of food skin, the direction of food fibers, cooking methods, and temperature differences change the physical properties of the food. Therefore, the composition and structure of the foods or eating habits of patients should be taken into consideration when conducting a food questionnaire survey.
